# Perioperative Radiographic Predictors of Non-Union in Infra-Isthmal Femoral Shaft Fractures after Antegrade Intramedullary Nailing: A Case–Control Study

**DOI:** 10.3390/jcm11133664

**Published:** 2022-06-24

**Authors:** Wei-Cheng Hung, Chin-Jung Hsu, Abhishek Kumar, Chun-Hao Tsai, Hao-Wei Chang, Tsung-Li Lin

**Affiliations:** 1 Department of Orthopedics, China Medical University Hospital, Taichung 404327, Taiwan; d24645@mail.cmuh.org.tw (W.-C.H.); d5983@mail.cmuh.org.tw (C.-J.H.); kumar@aior.co.in (A.K.); d7940@mail.cmuh.org.tw (C.-H.T.); d22067@mail.cmuh.org.tw (H.-W.C.); 2 School of Chinese Medicine, China Medical University, Taichung 404333, Taiwan; 3 Anup Institute of Orthopedics and Rehabilitation, Patna 800020, India; 4 Department of Sports Medicine, College of Health Care, China Medical University, Taichung 406040, Taiwan; 5 Graduate Institute of Biomedical Sciences, China Medical University, Taichung 404333, Taiwan

**Keywords:** infra-isthmal femoral shaft fracture, non-union, antegrade intramedullary nailing

## Abstract

Antegrade intramedullary (IM) nailing is the gold standard treatment for femoral shaft fractures; however, the non-union rate of infra-isthmal femoral shaft fractures is still high after antegrade IM nailing. This retrospective case–control study aimed to determine the association between perioperative radiographic factors and the non-union of infra-isthmal femoral shaft fractures after antegrade IM nailing. Univariate and multivariate analyses were used to evaluate the radiographic risk factors of non-union. Ninety-three patients were included, with thirty-one non-unions and sixty-two matched controls between 2007 and 2017. All were regularly followed up for 2 years. Receiver operating characteristic analysis revealed that a ratio of the unfixed distal segment > 32.5% was strongly predictive of postoperative non-union. The risk factors for non-union were AO/OTA type B and C (odds ratio [OR]: 2.20), a smaller ratio of the distal fragment (OR: 4.05), a greater ratio of the unfixed distal segment (OR: 7.16), a higher ratio of IM canal diameter to nail size at the level of fracture (OR: 6.23), and fewer distal locking screws (OR: 2.31). The radiographic risk factors for non-union after antegrade IM nailing for infra-isthmal femoral shaft fractures were unstable fractures, shorter distal fragments, longer unfixed distal fragments, wider IM canal, and fewer distal locking screws. Surgeons must strive to avoid non-union with longer and larger nails and apply more distal locking screws, especially for unstable, wider IM canal, and shorter distal fragment fractures.

## 1. Introduction

Femoral shaft fractures are common in orthopedic high-energy injuries. Their worldwide annual incidence ranges from 10 to 21 cases per 100,000 individuals [1,2]. For decades, antegrade intramedullary (IM) nailing has been the gold standard treatment for acute adult femoral shaft fractures. As the indications for nailing have expanded to include more complex situations, the rates of non-union of acute femoral shaft fractures after antegrade IM nailing have also increased to 4.1–12.5% [3,4,5,6,7]. Various studies have reported the risk factors of non-union; these include open fractures, unreamed antegrade IM nailing, bone loss, soft tissue interposition, open reduction, the distraction of the fracture site, insufficient fixation, smoking, nonsteroidal anti-inflammatory drug (NSAID) usage, and delayed weight-bearing [3,4,5].

Park et al. categorized femoral shaft fractures into supra-isthmal, isthmal, and infra-isthmal fractures [8]. The infra-isthmal region has a thin cortex and poor bone stock. The distal fragment of an infra-isthmal fracture is difficult to stabilize, and instability may develop after the nailing procedure. In the infra-isthmus femoral region, the mechanical stiffness of the bone–implant complex diminishes during metaphysis-diaphysis transition [9]. Kim et al. demonstrated the statistically significant differences in non-union between isthmal and non-isthmal fracture sites [10]. Yang et al. reported that a greater ratio of fracture site to isthmus diameter as a preoperative predictive factor was associated with a higher postoperative complication rate, such as non-union [11]. Watanabe et al. reported that distal fragments shorter than 43% of the femoral length are a risk factor for aseptic non-union in femoral nailing [12].

To the best of our knowledge, no studies have identified the factors for non-union in infra-isthmal femoral shaft fractures after antegrade IM nailing. We hypothesized the unstable fracture pattern, shorter length of the distal fragment, wider IM canal at the level of fracture, and insufficient distal fixation would predict non-union in such fractures. Therefore, the purpose of this retrospective case–control study was to evaluate the perioperative radiographic risk factors for non-union in infra-isthmal femoral shaft fractures.

## 2. Materials and Method

This single-center, retrospective case–control study was conducted at the department of orthopedic surgery in a tertiary healthcare hospital. We identified patients with infra-isthmal femoral shaft fractures (ICD-9-CM codes 821.20) who were treated with antegrade IM nailing via closed reduction surgery between June 2007 and June 2017. A total of 93 patients met the following criteria for study inclusion: (1) age > 20 years, (2) infra-isthmal fractures, (3) isolated unilateral fractures, (4) acute and close fractures, (5) fixation within 3 weeks, and (6) follow-up > 2 years after the surgery. We excluded cases in which bony union may have been influenced by the following factors: (1) multiple fractures, (2) combined femoral neck fractures, (3) segmental fractures, (4) periprosthetic fractures, (5) pathological fractures, (6) open fractures, (7) brain injury, (8) open reduction, and (9) postoperative infections.

Age- and body mass index-matched skeletally mature adults who underwent interlocking nailing for an infra-isthmal femoral shaft fracture during the same study period as the test patients were selected from the orthopedic registry and included in the union and non-union groups. The case–control ratio was set to 1:2. The infra-isthmal region was defined as the region between the upper border of the transepicondylar width of the knee joint and the isthmus [13]. Fellow-trained orthopedic surgeons performed all surgeries via the standard approach of a closed reduction with IM nailing.

Bony union can be defined by bridging callus formation in at least three of the four cortices (as seen on radiographs), absence of motion or pain during physiological stress to the fracture, or presence of full weight-bearing ability [14]. Radiological non-union was classified as hypertrophic or atrophic [15] and was defined by a lack of cortical continuity on more than two cortices, lack of bridging callus formation, persistent fracture lines, or absence of signs of progression to healing for 3 months.

**Figure 1:**
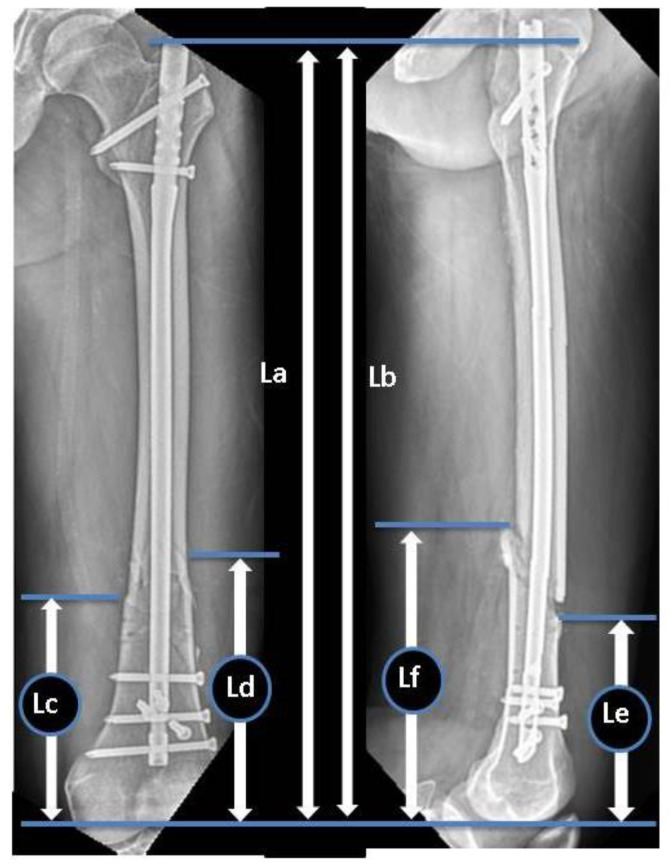
Ratio of the distal fragment: D/L, L: (La + Lb)/2, D: (Lc + Ld + Le + Lf)/4. D: Main distal fragment length, L: femur length, La: distance from the tip of the greater trochanter to the intercondylar notch in the AP view, Lb: distance from the tip of the greater trochanter to the intersection of the Blumensaat line and the trochlear groove line in the lateral view, Lc: distance from the intercondylar notch to the distal fracture line at the lateral site in the AP view, Ld: distance from the intercondylar notch to the distal fracture line at the medial site in the AP view, Le: distance from the intercondylar notch to the distal fracture line at the anterior site in the lateral view, Lf: distance from the intercondylar notch to the distal fracture line at the posterior site in the lateral view.

**Figure 2:**
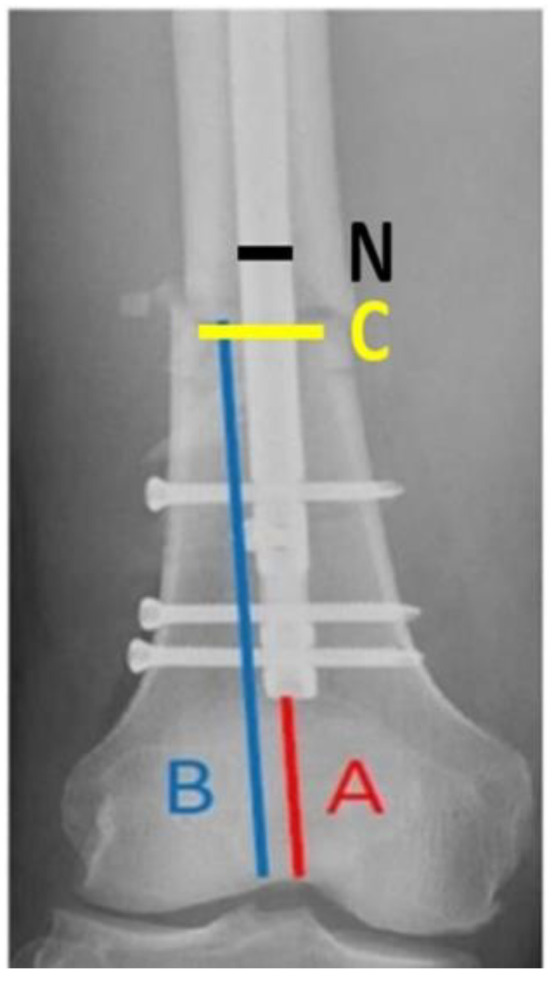
Ratio of the unfixed distal segment: A/B. A: Distance from the tip of the nail to the intercondylar notch in the AP view, B: main distal fragment length from the proximal fracture line to the intercondylar notch in the AP view. Ratio of the IM canal diameter to nail size at the level of fracture: C/N. C: IM canal diameter at the level of fracture in the AP view, N: nail size in the AP view.

During the study period, eight parameters were measured in the anteroposterior (AP) and lateral views of preoperative and immediate postoperative radiographs to evaluate the radiographic risk factors for non-union. Preoperative radiographs were evaluated to classify the fracture types in accordance with the AO Foundation/Orthopaedic Trauma Association (AO/OTA) classification system [16]. Goniometric measurements were performed using immediate postoperative radiographs to determine the coronal plane angulation on AP radiographs and the sagittal plane angulation on lateral radiographs (the long axes of the proximal and distal fragment’s diaphyses intersect). The ratio of the distal fragment was measured using immediate postoperative radiographs in accordance with the protocol by Watanabe et al. (Figure 1). The ratio of the unfixed distal segment, as well as the ratio of IM canal diameter to nail size at the level of fracture (C/N ratio) (Figure 2), and the number of distal locking screws and poller screws were also measured.

### Statistical Analysis

Statistical analyses were performed using IBM SPSS Statistics (version 23.0; SPSS Inc., Armonk, NY, USA). Descriptive statistics are presented as means and 95% confidence intervals (CIs) for continuous variables and as counts and percentages for categorical variables. The chi-squared test was performed for the nominal scale, while one-way analysis of variance was performed for the ratio scale. The areas under the curves were obtained using receiver operating characteristic (ROC) to predict the ratio of the unfixed distal segment and C/N ratio for postoperative non-union. Univariate and multivariate logistic regression analysis was performed for all variables to determine their relationship with the development of non-union after antegrade IM nailing. Statistical significance was defined as *p* < 0.05.

## 3. Results

**Figure 3:**
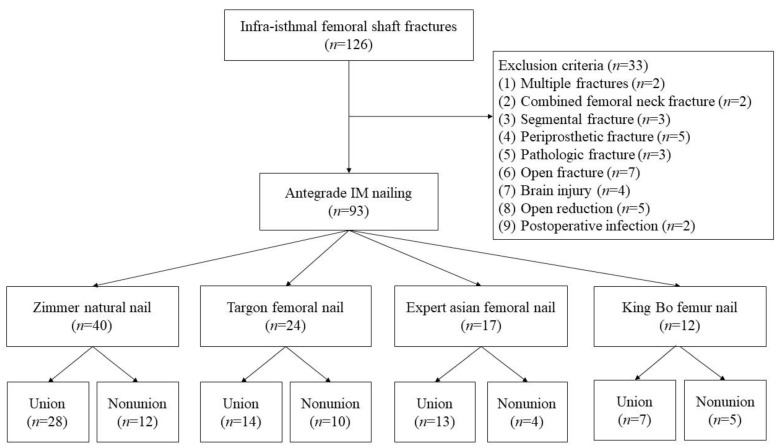
“Strengthening the Reporting of Observational Studies in Epidemiology” flowchart detailing the design of this study.

Ninety-three patients (70 men, 23 women) were included in this study. The mechanism of injury was high-energy trauma in all 93 patients. The following four types of antegrade nails were used in the treatment: (1) Zimmer Natural Nail (Zimmer-Biomet, Warsaw, IN, USA), (2) Targon femoral nail (Aesculap, Tuttlingen, Germany), (3) Expert Asian Femoral Nail (A2FN; Synthes, Solothurn, Switzerland), and (4) King Bo femur interlocking nail (Syntec Scientific Co, Changhwa, Taiwan). Figure 3 show the “Strengthening the Reporting of Observational Studies in Epidemiology” flowchart, which details the study design.

Based on the radiological classification, there were 25 hypertrophic non-union and 6 atrophic non-union cases. Bony unions took an average of 23.1 weeks to complete in the united cohort. The demographic and radiographic characteristics of the study participants are presented in Table 1.

**Figure 4:**
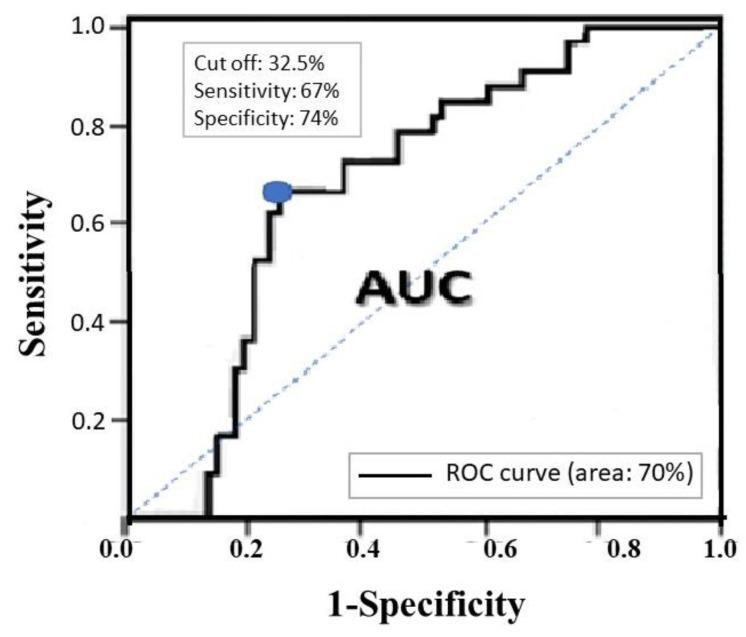
ROC curve for non-union. ROC analysis revealed that the summation of sensitivity and specificity was the greatest at the point indicated by the blue circle; it corresponds to a ratio of the unfixed distal segment that is 32.5% of the main distal fragment length.

A ratio of the unfixed distal segment > 32.5% was substantially predictive of postoperative non-union, according to ROC curve analysis (Figure 4). Furthermore, the ROC curve indicated that the C/N ratio > 2.1 had sensitivity and specificity of 69% and 70%, respectively, in predicting non-union.

Table 2 present univariate risk factors for the non-union of infra-isthmal femoral shaft fractures after antegrade nailing. On multivariate logistic regression analysis, AO/OTA classification types B and C, ratio of the distal fragment < 43%, ratio of the unfixed distal fragment > 32.5%, C/N ratio > 2.1, and distal locking screws < 3 were identified as independent risk factors for non-union after antegrade nailing (Table 3).

## 4. Discussion

This is the first retrospective case–control study to evaluate the perioperative radiographic predictors of non-union in infra-isthmal femoral shaft fractures after antegrade IM nailing; these included unstable fractures (AO/OTA classifications B and C), shorter distal fragments, longer unfixed distal fragments, wider IM canal, and fewer distal locking screws. Moreover, the most noteworthy finding of this study was that a ratio of the unfixed distal segment > 32.5% was substantially predictive of postoperative non-union.

For long bone fractures, retrospective studies revealed comminution and increasing displacement as the potential risk factors of non-union [17], while prospective studies confirmed the unstable fracture type as a significant risk factor for non-union [18]. In this study, we found that the AO/OTA classifications demonstrated a significant difference between the union and non-union groups. This may explain why the non-union rates depended on the unstable and comminuted fracture patterns.

The rotational instability of infra-isthmal fractures is due to the movements of the gastrocnemius and hamstring tendons that occur during motion. Furthermore, it is also attributed to the fact that the medullary cavity of the infra-isthmal fragment has a diameter that is substantially larger than that of the nail employed [10,19]. Thus, the infra-isthmal area of the femur is thought to be responsible for reducing the mechanical stiffness of the bone-implant constructs [9]. Yang et al. reported that the ratio of fracture site to isthmus canal diameter ≥ 2 was a reliable preoperative parameter to predict non-union [11]. In the current study, C/N ration > 2.1, as a postoperative parameter which revealed a significant difference between the union and non-union groups, was similar to Yang’s finding. This finding could explain why the wider IM diameter at the fracture site is related to a higher non-union rate after antegrade IM nailing.

Watanabe et al. noted that if the length of the distal fragment is less than 43% of the total femoral length, further surgical fixation can be considered [12]. Ha et al. reported that the instability of distal fragments leads to higher failure rates in non-union cases [20]. According to our results, the ratio of the distal fragment differed significantly between the non-union and union groups; this could partially explain why a smaller distal fragment ratio was the predictor of non-union in the current study.

The fixation strength is thought to be enhanced when the nail’s tip is as close to the Blumensaat line as possible [10]. The current study’s findings revealed a significant difference between the union and non-union groups when the ratio of the unfixed distal segment was over 32.5%. Furthermore, the ratio of the distal fragment and the ratio of the unfixed distal fragment predicted the development of non-union after antegrade nailing. These two predictors support the importance of nail length in the risk of non-union.

Multiple surgical tools can augment bone-implant stability in distal femoral fragments, such as poller screws and more distal locking screws. Bryan et al. discussed the effect of poller screws with retrograde nails on distal fragment stability [21,22]. Kim et al. reported that exchange nailing in infra-isthmal femoral shaft non-union with a more secure distal fixation, such as with poller screws and additional interlocking screws, may be an effective and reliable treatment option [10]. In the current study, the distal screw density was significantly different between the union and non-union groups, whereas the poller screws did not show any obvious intergroup differences. This suggests that increasing the distal screw density may be an effective way to reduce the likelihood of non-union.

There are several treatments for non-union of infra-isthmal femoral shaft fractures following an internal fixation; however, their outcomes remain debatable. Pihlajamäki et al. stated that exchange nailing without bone grafting was the best method for treating non-union after an index femoral nailing surgery [4]. Brinker et al. reported their experience of exchanging preexisting nails with nails that were at least 1 mm larger than the previous nails or up to 4 mm in size (if the previous nail was undersized) for treating non-union [23]. Park et al. reported that augmentation plating with autogenous bone grafting might be a better option than exchange nailing [8]. Various studies have reported that replacing the original nail with a retrograde nail can improve distal fragment stability [21,22]. Indeed, the advantages of retrograde IM nailing are a longer working length, adequate fixation of the distal fragment through the use of more interlocking screws, and easier reduction of the short distal fragment. Furthermore, Auston et al. reported that a retrograde IM nail combined with long segment blocking screws significantly increased stability by eliminating the “Bell-Clapper effect” in patients with distal femur fracture who had low bone quality, such as the elderly [24]. However, the disadvantages of this surgical method include cartilage damage to the femoral condyle and postoperative knee pain. Several studies have compared the functional outcomes and knee pain incidence between antegrade and retrograde nailing; however, these are currently inconclusive with debatable findings [6,25,26].

This study had several limitations. First, this was a retrospective case–control study with a limited number of patients and different surgeons, which could have led to some bias. Second, we only mentioned radiographic risk factors; other risk factors, such as smoking, effects of reaming, the timing of weight-bearing, and NSAID usage, were not considered. Third, four types of antegrade IM nail systems were utilized for fixation, giving rise to potential implant bias. Therefore, larger clinical studies are required to tackle these limitations.

## 5. Conclusions

The perioperative radiographic predictors of non-union in infra-isthmal femoral shaft fractures after antegrade IM nailing were unstable fractures, shorter distal fragments, longer unfixed distal fragments, higher C/N ratio, and fewer distal locking screws. A ratio of the unfixed distal segment > 32.5% is a reliable parameter for predicting instability and non-union after surgery. We recommended the use of longer and larger nails with more distal locking screws when treating infra-isthmal femoral shaft fractures, especially those with an unstable pattern, wider IM canal, and shorter distal fragments.

## Figures and Tables

**Table 1:** Study variables and their association with non-union and union of fractures.

	Non-Union (*n* = 31)	Union (*n* = 62)	*p*-Value
Age, years (95% CI)	28.6 (20–34)	28.2 (20–35)	0.687
Male, *n* (%)	23 (74.2)	47 (75.8)	0.672
Right laterality, *n* (%)	18 (58.1)	40 (64.5)	0.514
BMI, kg/m^2^ (95% CI)	23.6 (18.4–33.8)	24.1 (18.8–34.2)	0.761
AO/OTA classification			0.004
Type A, *n* (%)	11 (35.5)	42 (67.7)	
Type B and C, *n* (%)	20 (64.5)	20 (32.3)	
Postoperative coronal deformity, degree (95% CI)	7.0 (2–10)	6.7 (3–9)	0.341
Postoperative sagittal deformity, degree (95% CI)	3.6 (2–9)	2.8 (2–10)	0.412
Ratio of the distal fragment, % (95% CI)	34.6 (28–40)	44.5 (37–47)	0.021
Ratio of the unfixed distal fragment, % (95% CI)	32.3 (24.1–41.8)	23.8 (16.4–28.7)	0.013
C/N ratio, (95% CI)	2.2 (1.9–2.4)	1.9 (1.8–2.1)	0.028
Distal locking screws, n (95% CI)	2.1(2–4)	3.4 (2–4)	0.033
Poller screws, n (95% CI)	0.69 (0–2)	0.93 (0–4)	0.086

CI: confidence interval; *n*, number; BMI, body mass index; AO/OTA, AO Foundation/Orthopaedic Trauma Association; C/N, IM canal diameter to nail size at the level of fracture.

**Table 2:** Risk factors for non-union after antegrade nailing (univariate analysis).

Risk Factor	Odds Ratio (95% CI)	*p*-Value
AO/OTA classification type B and C	2.87 (0.67–5.84)	0.028
Ratio of the distal fragment < 43%	5.41 (2.09–10.65)	0.002
Ratio of the unfixed distal fragment > 32.5%	9.23 (3.44–24.06)	<0.001
C/N ratio > 2.1	8.71 (2.07–21.27)	0.001
Distal locking screws < 3	3.16 (1.94–9.17)	0.004

AO/OTA, AO Foundation/Orthopaedic Trauma Association; CI, confidence interval; C/N, IM canal diameter to nail size at the level of fracture.

**Table 3:** Independent risk factors for non-union after antegrade nailing (multivariate analysis).

Risk Factor	Adjusted Odds Ratio (95% CI)	*p*-Value
AO/OTA classification type B and C	2.20 (0.47–4.13)	0.037
Ratio of the distal fragment < 43%	4.05 (1.43–7.12)	0.018
Ratio of the unfixed distal fragment > 32.5%	7.16 (2.37–17.96)	0.031
C/N ratio > 2.1	6.23 (1.94–17.38)	0.017
Distal locking screws < 3	2.31 (1.08–6.18)	0.013

AO/OTA, AO Foundation/Orthopaedic Trauma Association; CI, confidence interval; C/N, IM canal diameter to nail size at the level of fracture.

## Data Availability

All the available data have been presented in this study. Details regarding data supporting the reported results can be requested at the following e-mail address: jeffrey59835983@gmail.com.
